# Mindfulness, rejection sensitivity and resilience in patients with thalassemia: a survey in Southeastern Iran

**DOI:** 10.3389/fpsyt.2025.1478217

**Published:** 2025-08-13

**Authors:** Asma Ghonchehpour, Batool Tirgari, Mahlagha Dehghan, Atefeh Ahmadi, Mansooreh Azizzadeh Forouzi

**Affiliations:** ^1^ Student Research Committee, Kerman University of Medical Sciences, Kerman, Iran; ^2^ Nursing Research Center, Kerman University of Medical Sciences, Kerman, Iran; ^3^ Reproductive and Family Health Research Center, Kerman University of Medical Sciences, Kerman, Iran; ^4^ Neuroscience Research Center, Institute of Neuropharmacology, Kerman University of Medical Sciences, Kerman, Iran

**Keywords:** mindfulness, rejection sensitivity, resilience, thalassemia, relationship

## Abstract

**Background:**

Thalassemia, due to its chronic nature, predisposes patients to various psychological issues, including rejection sensitivity, and can negatively impact resilience, thereby reducing quality of life. Mindfulness, as a psychological construct, has been shown to enhance emotional regulation and resilience, and may serve as a mediating factor in reducing rejection sensitivity and promoting resilience. This study aimed to investigate mindfulness and its correlation with rejection sensitivity and resilience in patients with thalassemia.

**Methods:**

This cross-sectional descriptive-correlational study involved 143 patients with thalassemia attending the Thalassemia Center in Kerman, Iran, during the year 2022-2023. Patients were asked to fill demographic and disease-related information form, the Freiburg Mindfulness inventory Short Form (FMI-SF), the Adult Rejection Sensitivity Questionnaire (A-RSQ), and the Conner-Davidson Resilience Scale. Data were analyzed using SPSS V.25.

**Results:**

The results showed a negative and significant correlation between mindfulness and rejection sensitivity (p = 0.001, r = -0.28). There was a positive and significant correlation between mindfulness and resilience (p<0.001, r = 0.48). Additionally, rejection sensitivity had a negative significant correlation with resilience (p < 0.001, r = -0.32). Linear regression analysis was used to assess the predictive relationships between variables. It indicated that mindfulness significantly predicted resilience (p < 0.05), while there was no significant predictive relationship between mindfulness and rejection sensitivity (p=0.05).

**Conclusion:**

This study found that mindfulness levels are associated with resilience and rejection sensitivity in patients with thalassemia. According to the results of the present study, tailoring interventions to effectively support thalassemia patients in managing rejection sensitivity and enhancing resilience could be beneficial, particularly when combined with mindfulness practices. However, further research is necessary to confirm their clinical effectiveness.

## 1 Introduction

Thalassemia is the most common chronic hereditary disease in the world (1–4). Approximately 240 million people worldwide are affected by Beta Thalassemia (4). In Iran, the prevalence of B-Thalassemia is around 4–8 births per 1,000 (5). This disease like, other chronic conditions, comes with various challenges (6). Its hereditary nature, onset in the early years of life, the likelihood of physical abnormalities, frequent visits for blood transfusions, chronic pain, and their impact on the growth, development, and family relationships of patients contribute to significant adverse effects on the mental health of both the patients and their family (4, 6, 7). The occurrence of acute psychological problems such as depression, anxiety, and mood disorders in patients with thalassemia can lead to psychological distress (3).

Mindfulness has its roots in Eastern meditative traditions and plays a central role in the teaching and philosophical framework of Buddhism. Mindfulness is recognized as a therapeutic approach for promoting mental health (8, 9). Kabat Zinn (2012) defines mindfulness as having awareness of present experiences (10). Mindfulness teaches individuals to accept negative thoughts instead of denying or rejecting them (11). Mindfulness brings about various positive psychological effects, including increased mental well-being, emotional acceptance, improved emotional regulation, a sense of vitality and joy, and reduced adverse mental symptoms (12).

Rejection Sensitivity is defined as the expectation of anxiety-provoking rejection in situations where the possibility of rejection by close individuals exists (13). The foundation of rejection sensitivity lies in attachment theories and cognitive-emotional processes (14, 15). Reinforcing anxious expectations in individuals who are sensitive to rejection, and the potential of feedback loops’ repetition, creates a dysfunctional cycle, leading the individual’s interactions with family, friends, strangers, choice of activities, hobbies, and interests; all under the influence of anticipated rejection and fear of it (15). Stability in rejection sensitivity reduces interpersonal relationships and is common in some personality/mental disorders (14). Depression, anxiety, and aggression are consequences of rejection sensitivity (16). Patients with thalassemia avoid attending social gatherings due to their physical problems, especially their appearance and bones, pale and yellow face, and show sensitivity towards the people around them and their interactions (3, 4, 14). Rejection Sensitivity appears in a person from the time of birth, and thalassemia disease is also with the patient from the time of birth, and with the growth of the child and increasing the child’s understanding of the disease and long-term treatments, it will be with him until the end of his life, and his rejection sensitivity will probably increase. It is assumed that rejection sensitivity originates from internal functional patterns that are based on experiences of rejection by the child’s caregivers (15).

According to the recent research findings, resilience reduction is among the challenges experienced by patients with thalassemia (3, 17). Resilience is defined as an individual’s capacity for successful adaptation, maintaining proper functioning, and preventing the recurrence of essential stressors in life (18). Resilience is recognized as a significant factor in preventing psychopathologies in vulnerable groups, aiding in coping with difficult and stressful life situations (19).

The results of Nasiri and colleagues’ study in 2015 aimed to evaluate the role of meaning in life, mindfulness and resilience in the flourishing of students showed that increasing mindfulness led to higher resilience, Furthermore, meaning in life and mindfulness showed an indirect effect on flourishing mediated by resilience (20). The findings of Huang and colleagues’ study in 2020, focusing on mindfulness, life skills, resilience, and emotional-behavioral problems in Chinese adolescents, indicated a statistically significant and positive relationship between mindfulness and resilience These results highlight the potential benefits of mindfulness and life skills education in strengthening resilience and lowering EBPs in gifted youth (21). The results of Liang and colleagues’ study in 2022 entitled “mindfulness and life satisfaction: the moderating effect of self-control and the moderated moderating effect of resilience”, showed that Resilience further influenced the moderating effect of self-control, such that, at high and moderate resilience levels, mindfulness and self-control together impacted life satisfaction (22).

Additionally, considering the importance of rejection sensitivity, intervention studies have been conducted on the resilience and mindfulness to address issues such as low levels of mindfulness, high levels of rejection sensitivity, and low resilience. In the study by Jabbarifard and colleagues in 2018, Mindfulness-Based Cognitive Therapy was found to be effective in increasing resilience among patients with major thalassemia also, in the study by Joss and colleagues in 2021, the mindfulness-focused intervention enhanced mindfulness, non-attachment, and empathy, leading to a decrease in interpersonal distress, rejection sensitivity, and various other psychological issues (17, 23). In the intervention study that was conducted on patients with thalassemia, it has been shown that the stress reduction intervention based on mindfulness had an effect in reducing the rejection sensitivity and increasing the resilience of these patients (24).

Regarding insufficient studies on the relationship between mindfulness, rejection sensitivity, and resilience in patients with thalassemia in Iran and worldwide, Given the high statistics of thalassemia in Iran and the aforementioned issues regarding problems such as rejection sensitivity and resilience, which are caused by psychological problems and can affect all aspects of the patient’s life and his family, the need to find non-pharmacological treatments is becoming more and more prominent day by day. The issue of mindfulness and its positive effects in various fields, including patients with thalassemia, is one of the issues that has received the attention of many researchers. Since there are not enough studies in the field of investigating the relationship between the three components of mindfulness and rejection sensitivity and resilience in patients with thalassemia in Iran and the world, this study was designed and implemented in Iran.

### 1.1 Study objectives

- To investigate the relationship between mindfulness, rejection sensitivity, and resilience in patients with thalassemia.
- To investigate the relationships between mindfulness, rejection sensitivity, resilience, and social demographics in patients with thalassemia.

## 2 Method

### 2.1 Study design and setting and key differences with previous publication research

This study is a descriptive-correlational study conducted on patients with thalassemia referring to the Thalassemia Center in Kerman, Iran in 2021-2022. The Thalassemia Center in Kerman provides services such as blood transfusions and specialized healthcare services for patients with thalassemia from Kerman city and its counties.

This current study builds upon and extends the findings of our previous publication “The effect of mindfulness-based stress reduction on rejection sensitivity and resilience in patients with thalassemia: a randomized controlled trial (24), while differing significantly in design, objectives, and methodology. The present study is a cross-sectional, observational study aimed at assessing the relationships between mindfulness, rejection sensitivity, and resilience among patients with thalassemia Unlike the previous study, which was an intervention-based trial utilizing Mindfulness-Based Stress Reduction (MBSR), no intervention was implemented in this study, and data were collected at a single time point. Regarding the sample, only 86 patients are similar to those in the previous study, while 57 patients differ from the prior sample. The data collection instruments are largely similar; both studies employed questionnaires to evaluate rejection sensitivity and resilience. However, the tool used to measure mindfulness in the current study is novel and was not utilized or assessed in the previous research. The analysis in this study emphasizes correlations and predictive relationships, whereas the previous study aimed to evaluate the effects of MBSR on reducing rejection sensitivity and enhancing resilience. Additionally, both studies were independently registered and approved by separate ethics committees at their respective research centers.

### 2.2 Sample size and sampling

310 patients with thalassemia were referred to the Thalassemia Center in Kerman. Morgan’s table was used to determine the appropriate sample size for a study. It is a commonly used approach in research design to estimate the sample size needed to make inferences about a population based on a representative sample. A total of 160 patients were included in the sample size calculation, factoring in a potential dropout rate of 192 individuals (Considering a 20% attrition rate). The sampling method in this study was convenient. Patients with thalassemia visited this center once every week or ten days, which may have limited our access to them. For this reason, we chose convenience sampling. The inclusion criteria for this study were as follows: participants had to be between 18 and 65 years of age, have a confirmed diagnosis of thalassemia major made by an oncology specialist (4), and self-report no known or treated psychological disorders (3, 4). The exclusion criteria included any participants who submitted incomplete questionnaire responses, specifically if more than 10% of the total items were unanswered (7). Additionally, individuals who were in an acute phase of their condition or newly diagnosed with thalassemia within the last six months were excluded from the study. the Researchers invited 200 patients to participate in the study, and 160 patients accepted to complete the questionnaires. 17 questionnaires were excluded, and 143 completed questionnaires were analyzed (response rate = 80%).

### 2.3 Data collection instruments

The instruments Included the demographic and disease-related information form, the Freiburg Mindfulness inventory Short Form (FMI-SF), the Adult Rejection Sensitivity Questionnaire (A-RSQ) and the Conner-Davidson Resilience questionnaires.

#### 2.3.1 Demographic and disease information

This questionnaire had two parts. The first part included demographic patients’ information, age, marital status, education level, gender, occupation, and number of children. The second part included disease-related information, such as thalassemia type, history of hospital stay (past 12 months), age at first blood transfusion, and a history of chronic illnesses.

#### 2.3.2 Short form of the Freiburg Mindfulness Inventory

The short form of the Freiburg Mindfulness Inventory (FMI-SF) was used to assess mindfulness in patients with thalassemia (25). Initially designed with 30 questions by Buchheld and colleagues in 2001, a short form (14 Items) was later developed by Walach and colleagues in 2006 (25). The short form effectively measures all aspects of mindfulness in individuals unfamiliar with Buddhist culture and mindfulness (26). Participants were required to respond to questions on a four-point Likert scale, ranging from rarely= 1 to almost always=4. Item 13 is reverse scored. The minimum score on this questionnaire is 14 and the maximum is 56, with higher scores indicating higher mindfulness. In a study conducted by Sauer and colleagues, the reliability coefficient for this questionnaire was found to be 0.88 (27). In Iran, Golpour and colleagues (2012) reported a Cronbach’s alpha coefficient of 0.73 confirming the reliability of the questionnaire (28). In the present study, the Cronbach’s alpha coefficient was obtained 0.79.

#### 2.3.3 Adult Rejection Sensitivity Questionnaire

To measure sensitivity to rejection, the Adult Rejection Sensitivity Questionnaire (A-RSQ), designed by Downey and Feldman in 1996 for assessing sensitivity to rejection, was used (29). This questionnaire normed in Iran (30), consists of two parts (A and B), with the participant requesting something from others in each situation, Respondents rate their anxiety and concern about the outcome, and their expectations regarding acceptance or rejection on a 6-point Likert scale from 1”Not concerned at all to 6 “ Very likely”. in part B participant Reponses to hypothetical situations questionnaire are based on two dimensions: “Anxiety and concern about non-acceptance of requests in partA, assessing the respondent’s anxiety in situation related to each question and part B, “expectation of response,” evaluation the likelihood of receiving a positive response from the other person. The degree of rejection sensitivity is calculated by first subtracting the scores in part B from the number 7, and then multiplying the expected likelihood of rejection for each situation by the degree of anxiety, so the mean score for 9 situations is obtained. The total score of rejection sensitivity will be the mean score of rejection sensitivity in 9 situations, ranging from 1 to 36, with higher scores indicating higher sensitivity to rejection (30, 31). The A-RSQ has reported Cronbach’s alpha coefficients of 0.90 and test-retest reliability of 0.91 in a study by Brenson et al. (2018) (32). In Iran, the questionnaire has demonstrated content validity in the study by Karamlou et al. (2015), with a reported Cronbach’s alpha coefficient of 0.81 (33). Also, in a study conducted on thalassemia patients in Iran, the Rejection Sensitivity Questionnaire was confirmed with a Cronbach’s alpha coefficient of 0.75 (24). In the present study, the Cronbach’s alpha coefficient was obtained 0.72.

#### 2.3.4 Conner-Davidson Resilience Scale

The Conner-Davidson Resilience Scale was used to measure resilience, designed by Conner and Davidson in 2003 (34). This questionnaire comprises 25 statements on a 5-point Likert scale from 0 (completely incorrect) to 4 (always correct). The score range is between 0 and 100, with higher scores indicating greater resilience. Conner-Davidson reported a Cronbach’s alpha coefficient of 0.90 for the resilience scale in American participants (34). In Iran, the resilience questionnaire has been normalized by Mohammadi et al. (2006), showing content validity and a reliability coefficient of 0.89 using Cronbach’s alpha (35). Also, in a study conducted on thalassemia patients in Iran, the Resilience Questionnaire was confirmed with a Cronbach’s alpha coefficient of 0.95 (24). In the present study, the Cronbach’s alpha coefficient was obtained 0.89.

### 2.4 Data collection

After obtaining the necessary permissions and approvals from the health authorities in Kerman city, the researcher initiated the data collection process. Sampling was conducted during the morning shift from April to mid-August 2022. The sampling of patients with thalassemia was done on a convenient. The researcher identified eligible individuals, invited them to participate in the study through individual interviews, explained the research objectives, execution method, and questionnaire filling process. Interested individuals were then requested to sign a written informed consent form and read the study details. After obtaining written consent, participants were enrolled in the study.

sampling occurred after the completion of healthcare services to ensure no disruption in the service delivery process, allowing participants sufficient time to respond to the questionnaire. Due to the patients’ compromised health status before blood transfusions, questionnaire completion was postponed until after the blood transfusion. Participants were assured that their participation would not affect their healthcare services, their information would remain confidential, and there was no need to disclose their full names. Subsequently, the questionnaire was provided to them, and they were asked to complete the demographic, mindfulness, rejection sensitivity, and resilience questionnaires. For participants with limited literacy, the questionnaire was read aloud.

### 2.5 Data analysis

Data analysis was performed using SPSS software version 25. Descriptive statistics, including frequency, percentage, mean, and standard deviation, were employed to summarize the demographic characteristics of the participants as well as the mean scores for mindfulness, rejection sensitivity, and resilience. Prior to conducting inferential analyses, the assumptions of normality, linearity, and homoscedasticity were assessed. Normality of continuous variables was evaluated using the Shapiro-Wilk test and visual inspection of Q-Q plots. Variables with p-values greater than 0.05 in the Shapiro-Wilk test were considered normally distributed. Linearity between variables was assessed through scatterplots. Homoscedasticity was examined by inspecting residual plots and conducting the Breusch-Pagan test. For inferential statistics, the Pearson correlation coefficient was utilized to assess the strength and direction of the relationships between the variables of interest. Simple linear regression analysis was conducted to determine the predictive relationships among mindfulness, rejection sensitivity, and resilience. To further explore the correlation between demographic variables (such as age, gender, and education level) and the scores for mindfulness, rejection sensitivity, and resilience, additional statistical tests were applied. Specifically, Pearson correlation was used to evaluate linear relationships, while independent t-tests were conducted to compare means between two groups. Analysis of variance (ANOVA) was also performed to assess differences in mean scores across multiple groups. Also, Bonferroni correction was used to reduce Type I error risk. A significance level of p < 0.05 was considered statistically significant.

## 3 Results

### 3.1 Socio-demographic characteristics

143 patients participated in this research with a mean age of 27.11 ± 5.44. 59.4% were female. 70% of the participants were single and 87.2% had no children. The education level of 45.5% of patients with thalassemia was at the diploma level. 37.2% were self-employed and 92.3% of the patients did not have any other chronic disease except thalassemia. The Bonferroni correction showed a significant difference between the marital status and the mean scores of mindfulness (p=0.02) and resilience (p= 0.03), the mean score of mindfulness and resilience in divorced patients was higher than in married patients. Also, resilience was higher in employed patients than unemployed patients (p= 0.04). There was no significant difference between other demographic variables and the mean scores of mindfulness, rejection sensitivity and resilience (p>0.05) (**Table 1**).

**Table 1:** The distribution of the participants demographic characteristics.

Variable	Frequency	Percent
Marital status		
Single	99	70.2
Married	36	25.5
Divorced	6	4.3
Children No.		
0	123	87.2
1	18	12.8
Age of the first transfusion		
2 months-one year	100	69.9
Above one year	43	30.1
History of hospital stay in the last year		
Yes	62	43.4
No	81	56.6
Education level		
Middle/high school	44	30.8
Diploma	65	45.5
Associate degree	11	7.7
Bachelor’s/higher	23	16
Sex		
Female	85	59.4
Male	58	40.6
Job		
Unemployed	45	32.8
Employed	9	6.6
Housewife	32	23.4
Self-employed	51	37.2
History of chronic diseases		
Yes	11	7.7
No	132	92.3

### 3.2 Mean scores of mindfulness, rejection sensitivity and resilience

**Table 2** indicates the mean scores of mindfulness, rejection sensitivity and resilience of patients with thalassemia major. The mean score of Mindfulness was 34.34 ± 7.52, the mean Rejection Sensitivity score was 9.85 ± 4.41, and the mean Resilience score was 64.99 ± 17.05 (**Table 2**).

**Table 2:** Mean (± SD) of mindfulness, rejection sensitivity and resilience in participants.

Variable	Mean ± SD
Total Mindfulness	34.34 ± 7.52
Total Rejection Sensitivity	9.85 ± 4.41
Total Resilience	64.99 ± 17.05

### 3.3 Correlation between the scores of mindfulness, rejection sensitivity and resilience

Prior to conducting Pearson correlation and linear regression analyses, all statistical assumptions were rigorously assessed. Normality of the data was confirmed using Shapiro-Wilk tests (*p* >.05) and visual inspection of Q-Q plots. Linearity was verified through scatterplots of variables, and homoscedasticity was examined using residual plots and the Breusch-Pagan test (*p* >.05). No significant violations of these assumptions were observed. **Table 3** shows the relationship between mindfulness, rejection sensitivity and resilience. There was a negative and significant correlation between mindfulness and rejection sensitivity (p=0.001, r=-0.28). There was a positive and significant correlation between mindfulness and resilience (p<0.001, r= 0.48). Also, rejection sensitivity had a significant negative correlation with Resilience (p< 0.001, r= -0.32) (**Table 3**).

**Table 3:** Correlation between Mindfulness, Rejection Sensitivity and Resilience.

Variable	Mindfulness	Rejection Sensitivity	Resilience
	Pearson Correlation		
Mindfulness	1	-0.28	0.48
P Value		0.001	< 0.001
Rejection Sensitivity	-0.28	1	-0.32
P Value	0.001		< 0.001
Resilience	0.48	-0.32	1
P Value	< 0.001	< 0.001	

### 3.4 Prediction

Also, Linear regression analysis was used to assess the predictive relationships between variables. It indicated that mindfulness significantly predicted resilience (β= 0.19, t = 8.17, p< 0.001, **R^2^** = 0.25, adjusted R^2^ = 0.24) while there was no significant predictive relationship between mindfulness and rejection sensitivity)p= 0.05) (**Table 4**).

**Table 4:** The relationship between mindfulness, rejection sensitivity and resilience.

Model	Unstandardized Coefficients		Standardized Coefficients	t	Sig.
	B	Std. Error	Beta		
1 (Constant)	19.31	6.97		2.77	0.007
Rejection Sensitivity	0.27	0.14	0.16	1.98	0.05
Resilience	0.20	0.04	0.45	5.50	<0.001

## 4 Discussion

Patients suffering from thalassemia may exhibit an increased risk of behavioral and psychological issues, along with a diminished quality of life. This study aimed to investigate the relationship between mindfulness, rejection sensitivity, and resilience in patients with thalassemia.

The study results showed a significant negative correlation between mindfulness and rejection sensitivity in patients with thalassemia. The results aligns with results of Ebrahimi (2023) research conducted on adolescents (36). Another study also reported a significant negative correlation between rejection sensitivity and mindfulness in young adults (37). Understanding the substantial negative connection between mindfulness and sensitivity rejection in this context requires exploring the nature of each concept. According to the theory of mindfulness and acceptance, mindfulness aids in observing momentary experiences, encouraging curiosity and interest rather than judgment, suppression, or avoidance (38). The negative relationship between rejection sensitivity and mindfulness in patients with thalassemia may stem from the difficulty in practicing mindfulness when individuals are preoccupied with concerns about rejection or negative evaluation. Heightened sensitivity to rejection might hinder the development or consistent practice of mindfulness techniques. The constant worry about being rejected can create a barrier to engaging effectively in mindfulness practices, and limit the potential benefits of managing emotional distress. Furthermore, mindfulness fosters adaptive responses to events instead of reactive responses. It helps individuals regulate their present-moment experiences, altering their interaction with emotional experiences when faced with rejecting events (38, 39). Consequently, mindfulness can shield individuals from sensitivity to rejection and maladaptive reactions following rejection incidents (40). Thus, the necessity of mindfulness interventions for individuals affected by thalassemia can impact their sensitivity to rejection and potentially enhance their interactions and quality of life.

The study findings indicate a significant positive relationship between mindfulness and resilience in patients suffering from thalassemia. No similar study has been found specifically on patients with thalassemia, findings from other populations align with those of the present study. The study by Nasiri et al. (2015) showed that enhancing mindfulness can lead to increased students resilience (20). Similarly, Huang et al. (2020) found a significant positive association between mindfulness and resilience in adolescents in China, where increased mindfulness correlated with higher resilience levels (21). Liang et al.’s research (2022) indicated that resilience’s moderating effect on mindfulness and self-control significantly impacted the enhancement of adolescents’ life satisfaction, linking resilience with mindfulness (22). The possibility of alignment between this study’s findings and others clarifies that individuals with mindfulness possess a different perspective compared to those without mindfulness. They tend to engage in more compassionate interactions and approach issues logically and realistically, which is expected to result in higher resilience levels. Badri et al. (2021) stated that mindful individuals exhibit greater resilience and are more open to new perceptual constructs, inclined toward increased creativity (41). Mindfulness transforms individuals’ perspectives on life, enhancing the quality of their relationships with themselves, their surroundings. Hence, mindfulness can potentially reduce negative psychological symptoms and enhance resilience (42).

The results showed a significant negative relationship between rejection sensitivity and resilience. Some studies have shown that at least 80% of individuals with thalassemia suffer from some form of psychological disorder. Emotional and psychological disorders in these patients can lead to decreased resilience and increased sensitivity to rejection. Additionally, the study findings also illustrated a significant negative correlation between resilience and sensitivity to rejection in the above-mentioned studies, including patients with thalassemia (43). Similarly, Schaan and Vogele’s study (2016) reported a significant negative correlation between resilience and sensitivity to rejection among individuals who had experienced divorce (44). Other study results predicted interpersonal sensitivity as a predictor of psychological resilience. Interpersonal sensitivity is equated with sensitivity to rejection is accompanying with validation seeking and significantly impacts psychological resilience. Higher scores regarding the need for validation indicate lower flexibility, leading to avoidance of social situations due to the negative evaluations, rejection, or other social circumstances (14, 43). High rejection sensitivity leads individuals to interpret their behaviors more negatively than others and associated with difficulties in interpersonal relationships (45).

The results showed that the mean score of mindfulness and resilience in divorced patients was higher than others. Our finding that divorced patients had higher mean scores of mindfulness and resilience can be interpreted as these individuals actively enhancing skills such as mindfulness and resilience to adapt to the new circumstances following the stress and crisis of divorce. Several studies have shown that mindfulness-based interventions and resilience training, especially among divorced women, lead to significant improvements in these traits and overall mental well-being Specifically, research confirms that mindfulness is directly associated with psychological well-being, and resilience plays a key mediating role in this relationship (46–48). Divorced individuals, facing multiple challenges, are more likely than others to develop and strengthen these skills to cope effectively. Also, resilience was higher in employed patients, which may be explained by the fact that employment provides individuals with a sense of purpose, financial independence, and social interaction, all of which contribute to enhancing resilience. Additionally, exposure to workplace challenges helps individuals develop coping skills and better manage daily stressors. Specifically, social and personal factors in the work environment, such as coworker support, participation in decision-making, and feeling valued at work, can increase resilience. Moreover, resilience enables employees to make better decisions during crises and maintain effective relationships with colleagues and patient (49, 50).

As thalassemia is a condition present from early life and carries distinctive features, it becomes a factor in generating negative emotions and subsequently leads to social relationship problems, further exacerbating sensitivity to rejection. For patients with thalassemia, practicing mindfulness can potentially offer tools to manage the distress associated with rejection sensitivity. Mindfulness practices aim to develop an individual’s ability to respond thoughtfully rather than react impulsively to distressing thoughts or emotions, fostering a sense of inner calm and self-compassion. Also encouraging positive coping mechanisms, promoting self-acceptance, and educating both patients and the community about thalassemia can enhance resilience. Empowering patients with knowledge about their condition and helping them develop a sense of belonging within supportive networks.

## 5 Limitation

One of the main limitations of this study is its cross-sectional design, which prevents establishing causal relationships between mindfulness, rejection sensitivity, and resilience. Therefore, the findings should be interpreted as associations rather than cause-and-effect conclusions. Additionally, the data were collected through self-report questionnaires, which may be subject to response biases such as social desirability or inaccurate self-assessment. Another limitation is the non-random, convenience sampling method used. Moreover, cultural and systemic factors unique to Iran, such as social norms, healthcare infrastructure, and psychological attitudes, might influence the observed relationships and limit the applicability of these findings to other cultural or national contexts. Future studies are encouraged to consider these cultural dimensions and replicate the research in different settings to assess the broader relevance of the results. we acknowledge that convenience sampling introduces potential selection bias, which may limit the representativeness of the sample.This limitation is important to consider when interpreting the results, and future research employing random sampling methods is recommended to enhance representativeness and reduce bias. Finally, to better understand the causal mechanisms underlying the relationships among mindfulness, rejection sensitivity, and resilience, experimental or longitudinal studies are recommended.

## 6 Conclusion

It is concluded that the higher rejection sensitivity, the lower is mindfulness and resilience. Higher the mindfulness, higher the resilience among patients with Thalassemia. While these results suggest potential relationships, further longitudinal or experimental studies are necessary to establish causality and to evaluate the effectiveness of mindfulness interventions in reducing rejection sensitivity and enhancing resilience. Nevertheless, our study provides a foundation for future research exploring these connections and developing targeted interventions. Looking ahead, future studies could explore the development and testing of targeted psychological interventions, such as mindfulness-based therapies, resilience training, or counseling programs, to examine their effectiveness in improving psychological well-being and adaptive coping among Patients with thalassemia. Longitudinal research is also needed to assess how these factors influence disease management and health outcomes over time. Clinically, these insights highlight the importance of integrating mental health support into Thalassemia care. Healthcare providers might consider implementing seminars, workshops, and psychoeducational programs focused on fostering mindfulness, resilience, and healthy lifestyle habits. Additionally, offering counseling and psychoeducational services could play a vital role in reducing rejection sensitivity and enhancing patients’ overall mental health. Ultimately, such comprehensive approaches could contribute to better psychological and physical health outcomes for individuals living with Thalassemia.

## Author’s note

All authors confirm that the following manuscript is a transparent and honest account of the reported research. This research is related to a previous study by the same authors titled “The effect of mindfulness-based stress reduction on rejection sensitivity and resilience in patients with thalassemia: a randomized controlled trial”. The previous study was performed on an investigation into the effect of a mindfulness intervention based on stress reduction on promoting resilience and reducing rejection sensitivity in patients with thalassemia, and the current submission focuses on investigating the relationship between mindfulness, rejection sensitivity, and resilience in patients with thalassemia. The study follows the methodology explained in the methodology section.

## Data Availability

The original contributions presented in the study are included in the article/Supplementary Material. Further inquiries can be directed to the corresponding author.
